# Phase I/II study of stereotactic body radiotherapy boost in patients with cervical cancer ineligible for intracavitary brachytherapy

**DOI:** 10.1007/s11604-024-01566-8

**Published:** 2024-04-16

**Authors:** Kei Ito, Yujiro Nakajima, Hiroaki Ogawa, Akiko Furusawa, Keiko Nemoto Murofushi, Satoshi Kito, Nao Kino, Toshiharu Yasugi, Takashi Uno, Katsuyuki Karasawa

**Affiliations:** 1 Division of Radiation Oncology, Department of Radiology, Tokyo Metropolitan Cancer and Infectious Diseases Center Komagome Hospital, 3-18-22 Honkomagome, Bunkyo-ku, Tokyo, 113-8677 Japan; 2 Department of Radiological Sciences, Komazawa University, 1-23-1 Komazawa, Setagaya-ku, Tokyo, 154-8525 Japan; 3 Department of Radiation Oncology, Tohoku University Graduate School of Medicine, 1-1 Seiryo-machi, Aoba-ku, Sendai, 980-8574 Japan; 4 Department of Gynecology, Shizuoka Cancer Center Hospital, 1007, Shimonagakubo, Nagaizumi-cho, Sunto-gun, Shizuoka, 411-8777 Japan; 5 Department of Gynecology, Tokyo Metropolitan Cancer and Infectious Diseases Center Komagome Hospital, 3-18-22 Honkomagome, Bunkyo-ku, Tokyo, 113-8677 Japan; 6 Diagnostic Radiology and Radiation Oncology, Graduate School of Medicine, Chiba University, 1-8-1 Inohana, Chou-ku, Chiba, 260-8677 Japan

**Keywords:** Brachytherapy ineligibility, Cervical cancer, Clinical trial, Phase I/II study, Stereotactic body radiotherapy boost

## Abstract

**Purpose:**

Stereotactic body radiotherapy (SBRT) boost is a promising treatment for cervical cancer patients who are ineligible for intracavitary brachytherapy (ICBT). The aim of this multicenter, single-arm, phase I/II study was to prospectively evaluate the efficacy and toxicity of SBRT boost.

**Materials and methods:**

ICBT-ineligible patients with untreated cervical cancer were enrolled. Patients underwent whole-pelvic radiotherapy (45 Gy in 25 fractions) with SBRT boost to the primary lesion. In the phase I dose-escalation cohort (3 + 3 design), patients were treated with SBRT boost of 21 or 22.5 Gy in three fractions. Although dose-limiting toxicity was not confirmed, a dose of 21 Gy was selected for the phase II cohort because it was difficult to reproduce the pelvic organs position in two patients during the phase I trial. The primary endpoint was 2-year progression-free survival.

**Results:**

Twenty-one patients (phase I, n = 3; phase II, n = 18) were enrolled between April 2016 and October 2020; 17 (81%) had clinical stage III–IV (with para-aortic lymph node metastases) disease. The median (range) follow-up was 40 (10–84) months. The initial response was complete response in 20 patients and partial response in one patient. The 2-year locoregional control, progression-free survival, and overall survival rates were 84%, 67%, and 81%, respectively. Grade ≥ 3 toxicity was confirmed in one patient each in the acute (diarrhea) and late (urinary tract obstruction) phases.

**Conclusion:**

These findings suggested that a SBRT boost is more effective than the conventional EBRT boost and can be an important treatment option for ICBT-ineligible patients with cervical cancer.

**Study registration:**

This study was registered at the University Hospital Medical Information Network Clinical Trials Registry (UMIN000036845).

**Supplementary Information:**

The online version contains supplementary material available at 10.1007/s11604-024-01566-8.

## Introduction

Pelvic External Beam Radiotherapy (EBRT), in combination with Intracavitary Brachytherapy (ICBT), is the standard treatment for localized cervical cancer [1]. According to the United States Surveillance, Epidemiology, and End Results Program, brachytherapy is associated with longer overall survival (OS) than EBRT boost [2]. Thus, ICBT is critical for improving outcomes in patients with cervical cancer. However, some patients are ICBT-ineligible owing to either poor general condition or residual bulky tumors after whole-pelvic radiotherapy (WPRT) or refusal to undergo the procedure [3].

Stereotactic Body Radiotherapy (SBRT) delivers ablative doses of radiation to tumors via extreme hypofractionation and is, therefore, a potential alternative treatment for ICBT-ineligible patients [4, 5]. A systematic review of case series with small sample sizes suggested a high local control rate for SBRT boost, at 91% [6]. Propensity-matched analysis of the National Cancer Database showed that the OS rate of patients treated with SBRT boost is not inferior to that of patients treated with brachytherapy [7]. Thus, SBRT is a promising approach based on retrospective data. However, owing to a lack of evidence for SBRT from prospective trials [6], this prospective phase I/II trial was conducted to assess the clinical outcomes of SBRT for ICBT-ineligible patients with locally advanced cervical cancer.

## Materials and methods

### Patients

The inclusion criteria were as follows: (i) pathologically confirmed cervical squamous cell carcinoma, adenocarcinoma, or adenosquamous cell carcinoma; (ii) clinical primary tumor (T) stage 1b1–3b disease; (iii) clinical lymph node (N) stage 0–1, and metastasis (M) stage 0–1 [para-aortic lymph node (PAN) metastases only] according to the UICC-TNM Classification, 8th edition; (iv) ineligibility for ICBT; (v) Eastern Cooperative Oncology Group performance status of 0–2; and (vi) no history of pelvic radiotherapy. Patients with adequate bone marrow (hemoglobin, ≥ 10.0 g/dL; leukocytes, ≥ 3,000/mL; and platelets, ≥ 100,000/mL), as well as normal renal and hepatic function (serum creatinine, < 1.5 mg/dL; bilirubin, < 1.5 mg/mL; aspartate/alanine aminotransferase, < 100 IU/dL; and creatinine clearance, > 10 mL/min), were included. Both Computed Tomography (CT) and Magnetic Resonance Imaging (MRI) were performed for staging. In cases of clinical stage III or higher, Positron Emission Tomography (PET)-CT was additionally employed. Patients with severe comorbidities, or other active cancers within the last 3 years, were excluded.

Patients with any of the following were ineligible for ICBT: (i) a primary tumor with a maximum diameter > 7 cm on T2-weighted MRI at initial diagnosis; (ii) comorbidities (e.g., dementia or uterine myoma [unable to insert a uterine sonde]); (iii) a vaginal wall that cannot be expanded using a small-sized Cusco’s vaginal speculum due to vaginal stenosis or uterine prolapse; (iv) refusal to undergo ICBT (the trial was offered only after the patients refused standard treatment); and (v) gynecologists’ and radiation oncologists’ judgment that ICBT was unsuitable for medical reasons (e.g., advanced age). For patients with bulky primary tumors > 7 cm at initial diagnosis, two-step registration was adopted at the pre-SBRT stage to exclude those eligible for ICBT after WPRT.

### Study design

This open-label, multicenter, single-arm, prospective phase I/II study evaluated the clinical outcomes of SBRT boost for cervical cancer. The primary endpoint of phase I was to determine the recommended dose according to the frequency of dose-limiting toxicity caused by SBRT. The primary endpoint of phase II was 2-year progression-free survival (PFS) according to the recommended dose. Secondary endpoints of phase II included tumor response, locoregional control, freedom from distant metastasis, OS, and non-hematologic adverse effects (AEs).

The study protocol was approved by the Ethical Review Board of each participating institution (approval number 2311 in a representative institution). The explanation of the present trial covered the insufficient evidence for the experimental treatment, highlighted the potential for reduced effectiveness, and emphasized the risk of serious toxicity. Written informed consent was obtained from all patients. The study was registered with the XXXX (XXXX) and was conducted in accordance with the Declaration of Helsinki.

### Procedures

Two SBRT doses (21 and 22.5 Gy in three fractions) were analyzed from the point of view of safety in phase I. The recommended dose was determined to be 22.5 Gy [8]. However, because it was difficult to reproduce the pelvic organs position in two patients during the phase I trial, we adopted the lower dose of 21 Gy and stricter organ dose constraints for safety.

Radiotherapy consisted of WPRT followed by SBRT. In the WPRT phase, the patients drank 500 mL of water over a period of 30 min (after voiding completely) as the pretreatment. The clinical target volume primarily (CTV1) consisted of the gross tumor volume, uterine cervix, uterine corpus, parametrium, vagina, and ovaries [9]. In addition, WPRT covered the regional nodes, including the common, internal, and external iliac nodes, presacral nodes, obturator nodes [10], and PANs (if PAN involvement was present). To create the internal target volume (ITV), a 5-mm margin was added to the whole cervix and the gross tumor, and an anisotropic margin (10-mm superior-inferior and anterior–posterior margins; 5-mm left–right margin) was added to the uterine corpus. A 5-mm margin was added to the CTV1 + ITV to create the planning target volume for WPRT (PTV1). WPRT was performed using intensity-modulated radiotherapy (IMRT) (TomoTherapy; Accuray Inc., Sunnyvale, CA, USA). The prescribed dose (PD) was 45 Gy in 25 fractions.

The planning goals were as follows: 90% of the PTV1 was to receive ≥ 95% (decrease to 90% allowed) of the PD (95% PD ≤ PTV1 D_90%_); 50% of the PTV1 was to receive 100–103% (decrease to 98% or increase to 105% allowed) of the PD (100% PD ≤ PTV1 D_50%_ ≤ 103% PD); and the maximum dose was to be ≤ 107% (increase to 115% allowed) of the PD (PTV1 D_max_ ≤ 107% PD). The PD and dose constraints used in this study are summarized in Supplementary Table. Megavoltage CT was performed in each fraction to check uterus movement.

Concurrent chemotherapy with cisplatin was recommended during WPRT, except for patients with clinical stage IB1 and IIA1 disease. The recommended regimen was five to six courses of weekly cisplatin (40 mg/m^2^; up to a maximum dose of 70 mg) [11].

**Fig. 1:**
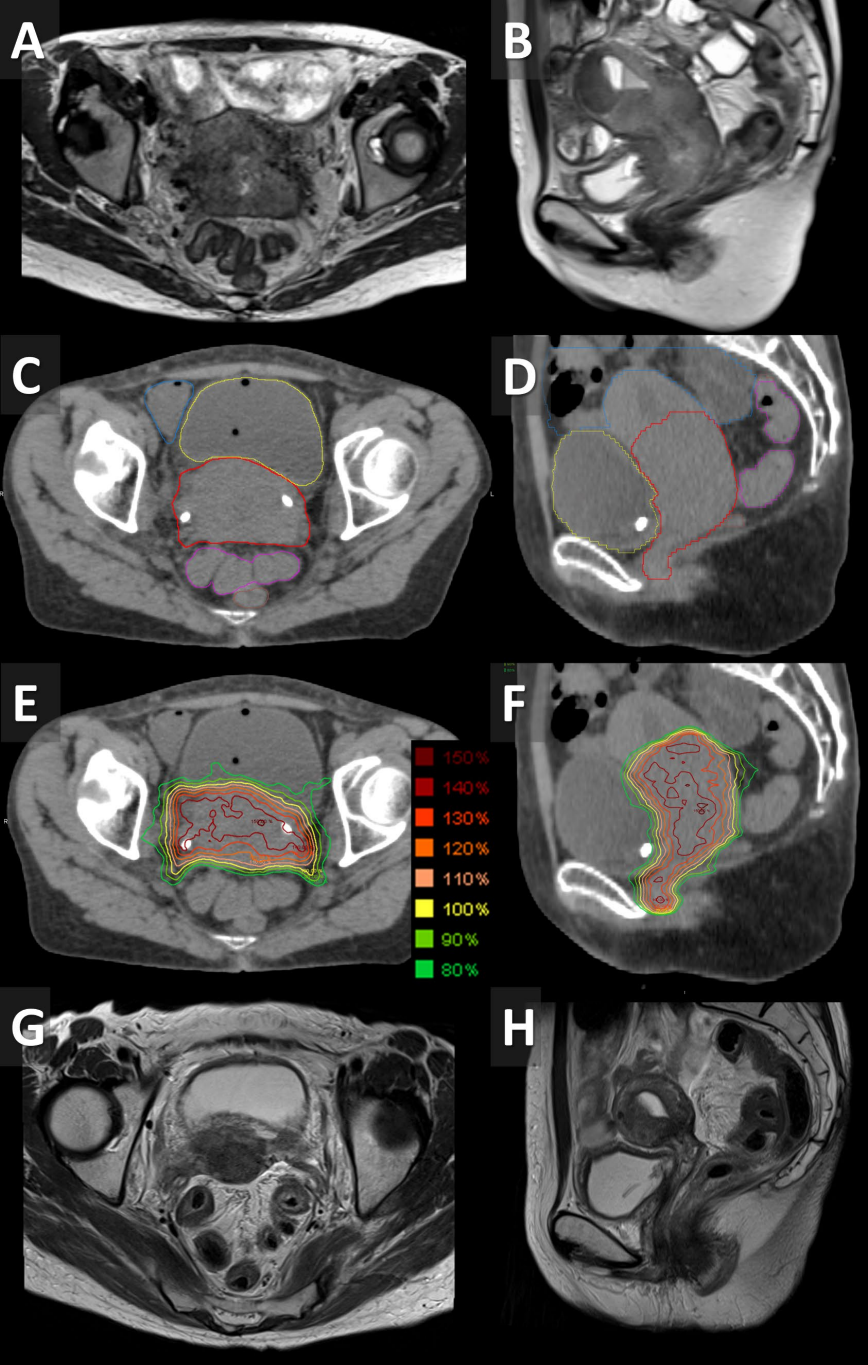
Images obtained from a 55-year-old woman with cervical cancer (T3bN1M0). **A** Axial and **B** sagittal T2-weighted MR images at SBRT initiation. **C** Axial and **D** sagittal CT images with contouring for planning SBRT (red = high-risk CTV). **E** Axial and **F** sagittal CT images with dose distribution of SBRT. **G** Axial and **H** sagittal T2-weighted MR images 3 months after SBRT. *CT* computed tomography, *CTV* clinical target volume, *MR* magnetic resonance, *SBRT* stereotactic body radiotherapy

During SBRT, a urinary catheter was placed, and the bladder was filled with 100–200 mL of physiological saline as pretreatment. Depending on the rectal volume, pretreatments, such as laxative administration, enemas, and rectal degassing with a catheter, were performed. No fiducial markers were inserted. After WPRT in 21 fractions, planning CT simulation was performed with a 1-mm slice thickness, and all patients underwent MRI for high-risk CTV (= CTV2) delineation (Fig. 1A, B). The CTV2 included the whole cervix, gross tumor, and suspected residual tumor at the time of SBRT (Fig. 1C, D) [12]. A 3-mm margin was added to the CTV2 to create the PTV for the SBRT (PTV2) to cover internal and set-up errors. Other organs at risk (OARs) were contoured using CT simulation images. A 3-mm margin added to the sigmoid colon and rectum was referred to as the planning OAR volume. SBRT consisted of a total dose of 21 Gy delivered in three consecutive daily fractions within 5 days (from Monday to Friday). The planning goals were as follows: 90% of the PTV2 was to receive ≥ 90% and ≤ 103% of the PD (90% PD ≤ PTV2 D_90%_ ≤ 103% PD), and the maximum dose was to be > 140% and ≤ 160% of the PD (140% PD < PTV2 D_max_ ≤ 160% PD) (Fig. 1E, F). Because the dose constraint for the OARs was prioritized, the permissible dose was 70% PD ≤ PTV2 D_90%_. Supplementary Table summarizes the dose constraints for the OARs. Patients received intramuscular scopolamine to control the creep motion immediately before irradiation. Treatments were delivered using IMRT with a 6-MV photon beam (Vero4DRT; Hitachi, Ltd, Tokyo, Japan and BrainLab AG, Munich, Germany). Treatment planning was performed with heterogeneity correction using the Monte Carlo algorithm in iPlan RT Dose (Brainlab AG, Feldkirchen, Germany) or collapsed cone algorithm in RayStation (RaySearch Laboratories, Stockholm, Sweden). Interfraction errors were corrected using six degrees of freedom, based on kilovoltage cone beam CT obtained once every 10 min during treatment delivery.

An IMRT boost of 9–14.4 Gy in five to eight fractions was directed against metastatic lymph nodes, according to tumor response to WPRT.

### Evaluation

Follow-up evaluations were performed 1 and 3 months after completing the treatment protocol and every 3 months thereafter for 2 years. CT and MRI were conducted alternately every 3 months for 2 years. PFS was calculated in months from the registration date to locoregional/distant failure, the most recent follow-up, or death. The initial tumor response was defined using MRI according to the Response Evaluation Criteria in Solid Tumours (version 1.1) [13]. Locoregional control was defined as the interval between the registration date and recurrence within the WPRT fields or the most recent imaging evaluation if tumors were controlled. Freedom from distant metastasis was defined as the interval between the registration date and recurrence outside the WPRT fields or most recent CT scan if tumors were controlled. OS was defined as the interval between the registration date and most recent follow-up or death from any cause. Non-hematologic AEs were evaluated according to the National Cancer Institute Common Terminology Criteria for Adverse Events (version 4) [14]. Acute AEs were defined as those occurring within 90 days after starting treatment; late AEs were those observed 90 days after starting treatment.

### Statistical analysis

A retrospective case series previously showed that the 2-year PFS rate after a conventional EBRT boost for ICBT-ineligible patients is 29% [3]. In the present study, the sample size calculation determined that 21 eligible patients would be required to test the 31% threshold for 2-year PFS with the expected value of 60% (based on prospective data on ICBT for locally advanced cervical cancer) [11], a one-sided significance level of 0.05, and a power of 80%.

PFS, locoregional control, freedom from distant metastasis, and OS were estimated using the Kaplan–Meier method. All analyses were performed using EZR software (version 1.54) [15], according to the intent-to-treat principle.

## Results

### Patient characteristics and treatment compliance

Although this was a two-center trial, 21 patients (phase I cohort, n = 3; phase II cohort, n = 18) were enrolled by a single institution for the phase II study between April 2016 and October 2020. All patients satisfied the inclusion criteria. The baseline characteristics are shown in Table 1. The median (range) age was 75 (49–91) years, and 17 patients (81%) had clinical stage III–IV disease. The median (range) primary tumor size was 59 (16–150) mm. Reasons for foregoing ICBT included bulky primary tumors (n = 7), comorbidities (n = 2), anatomical problems of the vagina (n = 2), patient refusal (n = 6), and “others,” (n = 4). “Others” included three older patients and one with a permanent contraceptive device. Among the nine patients enrolled due to their bulky primary tumors, three became eligible for ICBT as a result of WPRT; two were excluded during the two-step registration process, and one patient received SBRT because they refused ICBT.

**Table 1:** Patient characteristics

Characteristic		Patients, n = 21
Age, years	Median (range)	75 (49–91)
Clinical stage (UICC 8th)	IB/II/III/IV	3/1/14/3
Primary tumor stage	T1b/T2/T3a/T3b	4/2/3/12
Primary tumor size (mm)	Median/mean (range)	59/66 (16–150)
Lymph node stage	N0/N1	11/10
Para-aortic nodes	Negative/positive	18/3
Pathology	SCC	19
	ADC and SCC	1
	ADC	1
Reason for no ICBT	Bulky primary tumor	7
	Comorbidities	2
	Anatomical problem of the vagina	2
	Patient refusal	6
	Others	4

*ADC* adenocarcinoma, *ICBT* intracavitary brachytherapy, *SCC* squamous cell carcinoma, *UICC* the Union for International Cancer Control

All patients completed the treatment protocol without respite from radiotherapy. The median (range) treatment period was 43 (37–57) days. Owing to advanced age and patient refusal, chemotherapy was not administered concurrently in seven patients, although they had locally advanced lesions. Regarding dosimetric data, nine SBRT plans did not satisfy the desired dose to the target (90% PD ≤ PTV2 D_90%_) because priority was given to the dose constraints for the OARs. All plans satisfied the allowable dose (70% PD ≤ PTV2 D_90%_). All but one plan satisfied the dose constraints for the OARs; the rectal dose constraint was exceeded in one patient, in whom the primary tumor invaded the outer wall of the rectum. Seven patients received an IMRT boost for lymph node metastases, which remained after WPRT.

### Clinical outcomes

**Fig. 2:**
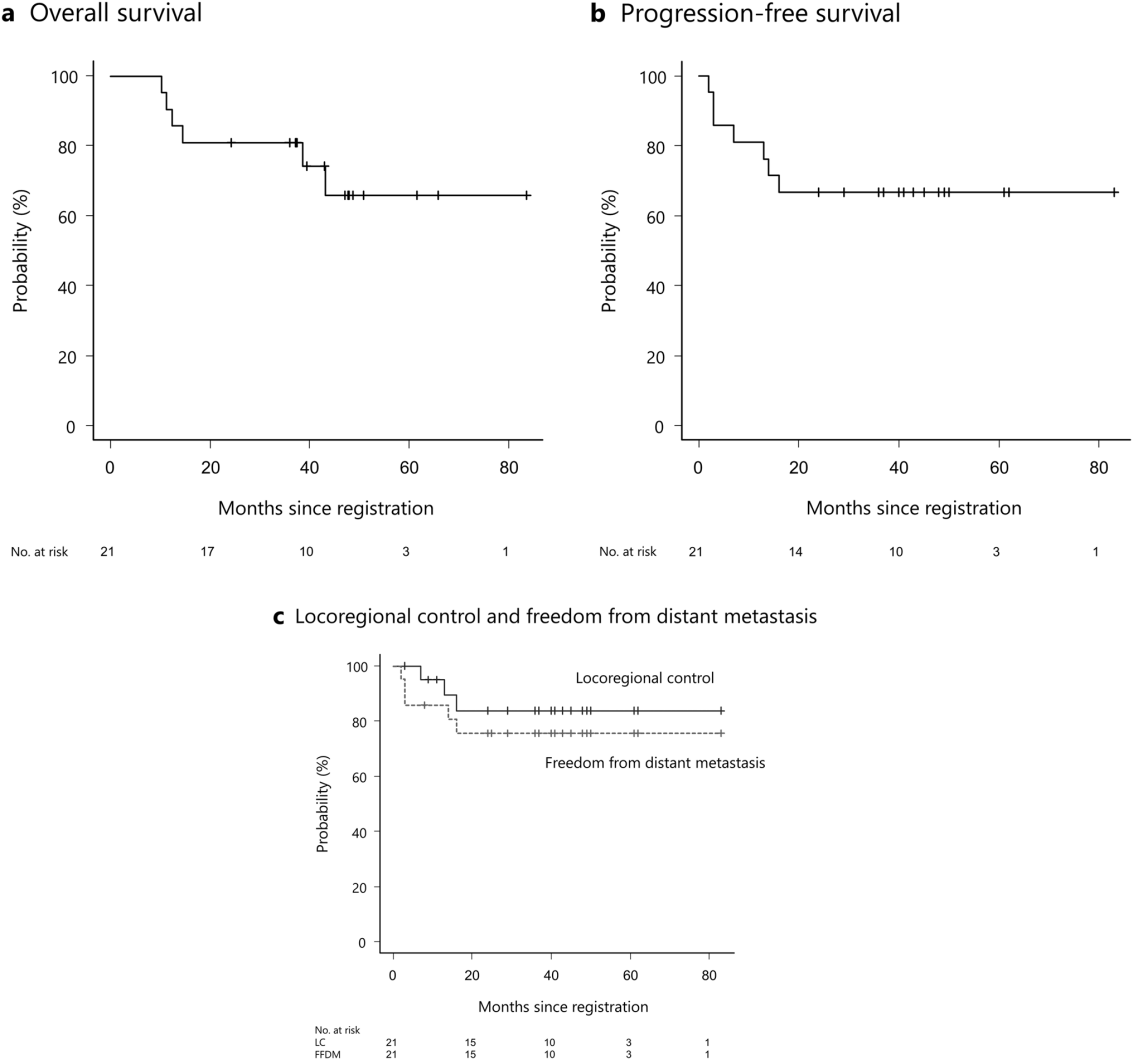
Kaplan–Meier curves of **A** OS, **B** PFS, and **C** locoregional control and freedom from distant metastasis. *OS* overall survival, *PFS* progression-free survival

The median (range) follow-up duration after registration was 40 (10–84) months. Six patients (29%) died at a median (range) of 13.5 (10–43) months owing to systemic disease progression. The 2-year OS rate was 81% (unreached median survival) (Fig. 2A).

The 2-year PFS rate was 67% (Fig. 2B). Regarding initial tumor response, complete and partial response was observed in 20 (95%) and one (5%) patients, respectively, although three primary lesions with no response or slight shrinkage in response to WPRT were included (Fig. 1G, H). The 2-year locoregional control rate was 84% (Fig. 2C). Local and regional lymph node recurrence was confirmed in three and zero patients, respectively. Local recurrence occurred in the center and margin of the SBRT field in two and one (rectal wall) patients, respectively. The 2-year freedom from distant metastasis rate was 76% (Fig. 2C). Three of five patients with distant metastasis had distant failure within 3 months of registration and had not received concurrent chemotherapy due to patient refusal or advanced age.

Table 2 summarizes the incidence of maximum AEs observed throughout the entire follow-up period. Grade ≥ 3 acute AEs were observed in one patient (Grade 3 diarrhea). Grade 2 or 3 late toxicities were observed in five (24%) and one (5% [urinary tract obstruction]) patients, respectively. No Grade ≥ 4 non-hematologic AEs were observed.

**Table 2:** Non-hematologic adverse effects

	Grade 2	Grade 3	Grade 4–5
Acute phase	6 (29%)	1 (5%)	0
Dermatitis	3	0	0
Diarrhea	3	1	0
Late phase	5 (24%)	1 (5%)	0
Proctitis	2	0	0
Urinary tract obstruction	0	1	0
Fracture	3	0	0

## Discussion

We conducted a phase I/II trial of SBRT boost in ICBT-ineligible patients with cervical cancer. SBRT boost achieved good locoregional control and PFS, despite the strong selection bias that seemed to negatively impact outcomes. Among five patients with distant metastases, three developed distant metastases within 3 months of registration. The three patients, therefore, may have had microscopic distant metastases before radiotherapy. These findings suggested the high curability of cervical cancer using SBRT in ICBT-ineligible patients. Moreover, the present results demonstrated that SBRT boost had very low toxicity.

A certain proportion of patients are ineligible for ICBT, as demonstrated by contraindications for ICBT in 14% of patients treated with definitive radiotherapy for locally advanced cervical cancer [3]. Some guidelines recommend interstitial brachytherapy (ISBT) [1, 16, 17] or conventional EBRT boost [18] in cases in which ICBT applicators fit poorly. ISBT can deliver a sufficient dose to irregularly shaped tumors while providing tolerable doses to the OARs, resulting in good local control [19]. However, ISBT is invasive, and not all ICBT-ineligible patients can undergo ISBT (e.g., patients with dementia and those who refuse invasive procedures). In contrast, conventional EBRT boost is applicable to all patients. However, conventional EBRT is difficult to administer at a high dose because of the dose constraints for the OARs, resulting in poor outcomes [2, 3, 20].

SBRT is a versatile approach and can be administered at high doses, thereby overcoming the limitations of ISBT and conventional EBRT boost. Approximately 36% of the experts globally indicated that a primary SBRT boost can be used when brachytherapy is contraindicated [5]; the current results supported this in terms of safety and efficacy. Conversely, a recent phase II trial of SBRT boost for cervical cancer reported a high incidence (27%) of Grade ≥ 3 rectal toxicity [21]. We considered reasons for these conflicting results by comparing these trials. The treatment protocols used in the two trials are summarized in Table 3. In the Albuquerque et al. [21] trial, SBRT used one additional fraction of radiation with the same 7 Gy per fraction. Additionally, their SBRT was not completed within 5 days (on weekdays) because the interval between fractions was ≥ 36 h. Therefore, it is possible that if the tumor shrank during the interval between the simulation CT for SBRT planning and final fraction of SBRT, the rectum may have been exposed to a higher dose than calculated. Albuquerque et al. [21] adopted 3 cc as the threshold for the rectal dose constraint (2 cc is commonly used in brachytherapy); their dose constraint was approximately 30 Gy (an equivalent dose at 2 Gy [EQD2]) higher than that used in the present trial. Consequently, the median and maximum irradiated rectal doses for 2 cc in the two trials differed by 20 (EQD2) and 30 Gy (EQD2), respectively, with the lower values having been used in our study. Besides, the rectal dose of the present study indicated the dose for the planning OAR volume of the rectum, whereas the rectal dose of the Albuquerque et al. [21] trial indicated that of the rectum itself.

**Table 3:** Comparison of two trials

	Treatment phase	Albuquerque et al. (2020)	Present study
Median CTV2 (range)	SBRT	82 (30–165) cc	58 (23–277) cc
Median PTV2 (range)	SBRT	139 (51–268) cc	79 (36–810) cc
Protocol dose of PTV. α/β = 10	WPRT	D_95%_ 45 Gy/25 fx	D_50%_ 45 Gy/25 fx
	SBRT	28 Gy/4 fx (interval ≥ 36 h)	D_90%_ 21 Gy/3 fx (every day)
	Total dose [EQD2]	83.9 Gy	74.0 Gy
Dose gradient inside PTV2	SBRT	Homogeneous	Maximum dose > 140% prescribed dose
Median PTV D_90%_ irradiated (range). α/β = 10	WPRT	NA	45 Gy*
	SBRT	NA	19.3 (16.4–23.1) Gy
	Total dose [EQD2]	NA	70.7 (65.4–78.3) Gy
PRV for the rectum	SBRT	None	3 mm
Dose constraint of rectum D_2 cc_. α/β = 3	WPRT	D_3 cc_ 45 Gy	45 Gy
	SBRT	D_3 cc_ 28 Gy	16.5 Gy
	Total dose [EQD2]	D_3 cc_ 99.2 Gy	71.3 Gy
Median rectum D_2 cc_ irradiated (range). α/β = 3	WPRT	NA	44.7 (44.0–44.9) Gy
	SBRT	NA	16.3 (14.5–16.9) Gy
	Total dose [EQD2]	90.6 (67.4–101.5) Gy	70.0 (65.3–71.9) Gy

*CTV* clinical target volume, *D*_*90%/2 cc*_ dose irradiated to the 90%/2 cc, *EQD2* equivalent dose at 2 Gy, *PRV* planning organs at risk volume, *PTV* planning target volume, *SBRT* stereotactic body radiotherapy, *WPRT* whole-pelvic radiotherapy

* Estimated value

The optimal dose fractionation of SBRT boost for cervical cancer remains unknown. In a survey, a panel of global experts selected the PD of SBRT boost with a wide range from 10 Gy in two fractions (12.5 Gy [EQD2]) to 40 Gy in five fractions (60 Gy [EQD2]) [5]. Although the PD should ideally be set to the same dose as the curative dose of brachytherapy (a total of 80–90 Gy [EQD2]) [1], delivering the dose for SBRT is often difficult owing to differences in patient cohorts, dose gradients, and planning margins. Therefore, we adopted a total of 74 Gy (EQD2) based on the total radiation dose of definitive radiotherapy with ICBT for Japanese patients with cervical cancer [22]. The reasons for the good outcomes despite the lower dose in this study may be as follows: (i) our SBRT delivered escalated doses to the central region of the primary lesion as well as ICBT; (ii) the highest possible dose was administered to the PTV overlapping the planning OAR volume, while satisfying the dose constraints for OARs; and (iii) the set-up error may be smaller than the setting, and the tumor received a higher dose than calculated.

The present study highlights the potential of SBRT as a new treatment option for ICBT-ineligible patients with cervical cancer. We anticipate that the number of patients requiring SBRT for cervical cancer will increase in the future. The low burden of SBRT may make it suitable for vulnerable and frail older patients [23] and patients with metastatic cervical cancer [24, 25]. In both cases, (semi) radical local treatment with the utmost consideration for safety and minimal patient burden is required. We are planning to conduct prospective trial in older adult patients to establish SBRT as a second option for cervical cancer.

This study has some limitations. First, the primary endpoint was PFS, despite the heterogeneous study population. Because the trial was limited to ICBT-ineligible patients, not all patients had poor prognosis (e.g., clinical stage I–II disease accounted for 19% of patients). Second, the desired planning goal (PTV2 D_90%_ ≥ 90% PD) was not satisfied in nine SBRT plans. Third, toxicities were graded based on medical interviews and examinations conducted by physicians. As a result, AEs, particularly mild toxicities like Grade 2, might have been underestimated. Fourth, the single-institutional setting of the study limits its generalizability. Radiation oncologists may find it challenging to appropriately perform SBRT boost for cervical cancer. Therefore, it is necessary to confirm the reproducibility of this study in other centers.

## Conclusions

To the best of our knowledge, this is the first phase II study completed pertaining to SBRT boost for cervical cancer. Our findings showed that SBRT resulted in good tumor control with less toxicity and suggested that SBRT boost may be an important option for ICBT-ineligible patients with cervical cancer. The irradiation methods used in this study may be useful in the absence of options besides SBRT boost in daily clinical practice.

### Supplementary Information

Below is the link to the electronic supplementary material.

Supplementary file1 (DOCX 26 KB)
