# Supplementary material for: Phase I/II study of stereotactic body radiotherapy boost in patients with cervical cancer ineligible for intracavitary brachytherapy
Source: Jpn J Radiol. 2024 Apr 16;42(8):909–17. doi: 10.1007/s11604-024-01566-8 (PMC11286639; doi:10.1007/s11604-024-01566-8)
Supplement: Supplementary file 1 — Supplementary file1 (DOCX 26 KB) [file 11604_2024_1566_MOESM1_ESM.docx]

**Supplementary Table.** **Prescribed dose and dose constraints**

| Each radiation dose | PTV D_90%_  α/β = 10 | Small bowel D_2 cc_  α/β = 6 | Bladder D_2 cc_  α/β = 3 | Sigmoid* D_2 cc_  α/β = 3 | Rectum* D_2 cc_  α/β = 3 |
| --- | --- | --- | --- | --- | --- |
| WPRT (EQD2)  SBRT (EQD2)  Total dose, EQD2 | 45 Gy in 25 fx (44.25 Gy)  21 Gy in 3 fx (29.75 Gy)  74.0 Gy | 45 Gy (43.9 Gy)  15 Gy (20.6 Gy)  64.5 Gy | 45 Gy (43.2 Gy)  22.5 Gy (47.3 Gy)  90.5 Gy | 45 Gy (43.2 Gy)  16.5 Gy (28.1 Gy)  71.3 Gy | 45 Gy (43.2 Gy)  16.5 Gy (28.1 Gy)  71.3 Gy |

D_2 cc/90%_ = dose irradiated to the 2 cc/90%; EQD2 = equivalent dose at 2 Gy; PTV = planning target volume; SBRT = stereotactic body radiotherapy; WPRT = whole-pelvic radiotherapy.

* The dose constraints were applied to the volume that a 3-mm margin was added to the sigmoid colon and the rectum.
